# Current status and perspectives of the quality system in histocompatibility laboratories in Poland

**DOI:** 10.3389/fgene.2024.1322414

**Published:** 2024-01-26

**Authors:** Izabela Uhrynowska-Tyszkiewicz, Katarzyna Bogunia-Kubik, Monika Baj-Krzyworzeka, Barbara Piątosa

**Affiliations:** ^1^ Department of Transplantology and Central Tissue Bank, Center of Biostructure Research, Medical University of Warsaw, Warsaw, Poland; ^2^ National Center for Tissue and Cell Banking, Warsaw, Poland; ^3^ Laboratory of Tissue Immunology, Medical Center, Hirszfeld Institute of Immunology and Experimental Therapy, Polish Academy of Sciences, Wroclaw, Poland; ^4^ Department of Clinical Immunology, Medical College, Jagiellonian University, Cracow, Poland; ^5^ Tissue Typing Laboratory, Department of Clinical Immunology, University Children’s Hospital of Cracow, Cracow, Poland; ^6^ Histocompatibility Laboratory, Children’s Memorial Health Institute, Warsaw, Poland

**Keywords:** HLA, histocompatibility, laboratories, Poland, transplantation, quality

## Abstract

Allogeneic transplantation is a multi-step process involving many clinicians and laboratory personnel working together to achieve a common goal—to maximize the recipients’ chance of survival and to improve their quality of life. One of the key elements of the process is to ensure high quality, accuracy, and reliability of histocompatibility testing. This manuscript presents: the development and organizational principles of the national system of supervision and control of histocompatibility laboratories in Poland, problems faced by these laboratories, availabe proficiency testing schemes, as well as suggestions and prospects for the future raised by members of the Polish histocompatibility community.

## Introduction

Many factors determine the success or failure of allogeneic transplantation of vital cells, tissues or organs. This type of transplantation involves a significant burden on the recipient’s immune system. An optimal immunological match between the recipient and a potential donor increases the chance of obtaining well-functioning allografts over a long period of time.

The principles of immune matching depend on the type of cells, tissues or organs to be transplanted, with different algorithms of matching for hematopoietic stem cell transplantation, vascularized organs, and multi-tissue transplants. Biostatic transplants and non-vascularized grafts usually do not require matching between the recipient and the donor.

Regardless of the selected algorithm, it is usually necessary to determine major histocompatibility complex (HLA) antigens of the patient from the waiting list and of the potential donor, with the scope of loci depending on the type of the allograft and requirements of the respective national transplantation program. In certain situations, monitoring of the immunological status of both the potential and actual recipient is also carried out.

Here we describe the development and organizational principles of the national system of supervision and control over histocompatibility laboratories in Poland, proficiency testing schemes, problems faced by these laboratories, as well as suggestions and prospects for the future raised by members of the Polish histocompatibility community. This description is based on publicly available data, data from control reports at Histocompatibility Laboratories (hereafter referred to as HcL), and data obtained from surveys returned by HcL.

### Histocompatibility laboratories (HcL) in Poland

In Poland, laboratory procedures in the field of transplantation immunology are performed in medical diagnostic laboratories, traditionally referred as Histocompatibility Laboratories (HcL). The nineteen laboratories involved in transplant immunology in Poland are part of various parent entities with a different legal status: regional blood centers, hospitals established by regional municipalities, university hospitals, and research institutes. HcL can be stand-alone units within the parent entity or be part of medical laboratories with a broader scope of activities.

The scope of tests performed by HcL depends on the involvement of an individual HcL in specific transplantation programs and/or programs for recruiting potential donors, especially potential unrelated hematopoietic stem cell donors, the type of parent entity, as well as contracts with healthcare providers.

Polish HcL use a wide range of techniques: HLA typing by molecular techniques at a low, high, and allelic resolution, anti-HLA antibody screening and identification, determination of PRA (panel reactive antibodies), anti-C1q antibody presence, and cross-matching, the latter also with the use of flow cytometry. Serological HLA typing is currently used only by two laboratories in parallel with molecular techniques: one laboratory uses it for HLA-B27 testing and one performs class I tests as an auxiliary method for evaluation of deceased donor cells’ reactivity in complement dependent tests.

### Organizational principles of the national system of HcL supervision and control

In Poland, transplantation immunology laboratories are supervised by dedicated institutions appointed by the Minister of Health (hereafter referred to as MoH) and, unlike other laboratories, are also directly responsible to MoH. For the past 17 years, Polish HcL must have complied with requirements of legal acts applicable to all medical laboratories (hereafter referred to as the general Laboratory Law) and additional requirements defined in the so-called Transplantation Law dated 2005 (Tinyurl, 2023a) implementing the provisions of European Union 2004/23/EC (Data Europa, 2004) and 2010/45/EU (Data Europa, 2010) directives, which came into force in January 2006. Moreover, every Polish medical laboratory must comply with common legal requirements relating to the medical profession, documentation, healthcare, patient rights, *etc.*

The general Laboratory Law details the requirements relating to personnel, premises, laboratory equipment, obligatory participation in external proficiency schemes, as well as pre-laboratory and laboratory activities that every medical laboratory must meet. None of these require *expressis verbis* the need to develop, implement, maintain, and continuously improve the quality management system (hereafter referred to as QMS) in laboratories or penalize non-adherence. Obtaining confirmation that the laboratory and/or the parent entity meet the requirements of ISO 9001:2015, 15189:2022 and/or 17025:2017, is voluntary in Poland. Certification regarding compliance with standards developed by professional societies such as European Federation for Immunogenetics (EFI) or American Society for Histocompatibility and Immunogenetics (ASHI) is also voluntary. The requirement for HcL to have a QMS is, on the other hand, included in the Transplantation Law.

Polish medical laboratories are currently in the transitional period with regard to the requirements of the general Laboratory Law. In December 2022, the Laboratory Medicine Act (Tinyurl, 2022a) came into force, replacing the Laboratory Diagnostics Act (Tinyurl, 2022b). Until December 2023, a number of implementing acts issued under the Laboratory Diagnostics Act are still in effect. Supervision over personnel in all laboratories, including HcL, is carried out by a dedicated body of self-government, the National Board of Laboratory Diagnosticians (Polish: KRDL). KRDL issues the laboratory diagnostician’s license, maintains a register of laboratory diagnosticians, and is responsible for keeping records of all health care-related laboratories, including HcL.

Additional supervision over HcL activities is also exercised directly by MoH who, under the Transplantation Law (Tinyurl, 2023a), grants permission to operate valid for 5 years from the moment it is granted. The procedure for obtaining such a permission is a multi-step process, currently free of charge to the applicant. Submitting an application describing the laboratory and its quality system is the first step in this process. Since 2009, the application is submitted to a dedicated entity, the so-called National Centre for Tissue and Cell Banking (Polish: KCBTiK). KCBTiK is a budgetary unit responsible to the Polish MoH, obliged to supervise tissue and cell banks, as well as to organize training in the field of procurement, testing, processing, sterilization, storage, and distribution of cells and tissues.

Following verification of the submitted application by KCBTiK, MoH conducts a control at HcL or orders it to be conducted. Such controls are carried out by MoH-authorized KCBTiK staff and sometimes by external experts designated by MoH in cooperation with the National Clinical Immunology Consultant. The ministerial control includes verification that the HcL in question meets both the requirements of the general Laboratory Law and the Transplant Law (**1**) and covers all areas of a given laboratory’s operations (Supplementary Table S1). In principle, controls are carried out at an HcL’s premises by two persons authorized by MoH.

During the COVID-19 pandemic, controls were carried out remotely. Regardless of the form of control, all activities are documented using a form developed by KCBTiK, based on the deliverables of the EU-funded project EUSTITE (“European Union Standards and Training in the Inspection of Tissue Establishments”).

The report prepared after the control may include certain post-control recommendations. Following verification that the HcL meets all legal requirements, KCBTiK submits an application to MoH for permission to perform specific tests, and with specific research techniques. MoH consults the application with the National Transplant Board and decides whether or not to grant permission, which is an administrative decision within the meaning of the Polish Code of Administrative Procedure (Tinyurl, 2023b). Critical non-conformities defined as imposing significant risk to patient health or life result in suspension of the permission to operate until effective corrective measures are implemented.

Legal requirements for HcL to obtain MoH permission to conduct its activities are presented in Supplementary Table S2.

All allotransplantation procedures in Poland are coordinated by a government agency POLTRANSPLANT, a budgetary unit subordinate to the MoH. POLTRANSPLANT maintains a number of transplant registries: the Central Registry of Objections, the National Transplant Waiting List, the Registry of Unrelated Potential Donors of Hematopoietic Stem Cells and Cord Blood, and the Transplant Recipient Registry. POLTRANSPLANT is also, along with the National Health Fund and the Ministry of Health, one of the payers for a specific catalog of such procedures.

### Proficiency testing schemes

All medical laboratories in Poland, including HcL, are required to participate in internal and external quality control schemes (hereafter referred to as EPT) to assess the qualifications of the laboratory personnel and quality of histocompatibility testing. The HLA Proficiency Testing for Central and East Europe (formerly the Proficiency Testing of HLA class I Typing for Central and East Europe) was the first attempt at EPT initiated in the early 1990s by Prof. Andrzej Lange (Bogunia-Kubik et al., 2000). Currently, this EPT is organized by the Hirszfeld Institute of Immunology and Experimental Therapy, Polish Academy of Sciences in Wroclaw under the auspices of the Polish Society for Immunogenetics. It is supervised and directed by Prof. Katarzyna Bogunia-Kubik, who also serves as regional coordinator for EFI Region 5. This EPT scheme is the only Polish EPT provider, and has been serving Polish standardization system for over 20 years.

Since 1999 when the first round of EPT has been organized, it been has extended to the wider Central-Eastern European area (Bogunia-Kubik and Lange, 2004; Bogunia-Kubik et al., 2006; Bogunia-Kubik and Lange, 2008; Bogunia-Kubik and Lange, 2009; Bogunia-Kubik, 2019). In total, 67 HcL from 16 countries, also from outside of Central and Eastern Europe, participated in Wroclaw EPT (Bogunia-Kubik, 2019). Over the years, the scheme has significantly evolved with respect to both the clinical material provided and number of HLA loci to be tested (Bogunia-Kubik, 2019), covering serological typing of HLA class I loci, as well as DNA typing of 11 HLA loci (A, B, C, DRB1, DRB3/4/5, DQB1, DQA1, DPB1, and DPA1).

The participants are provided with blood and/or DNA samples to test HLA class I antigens by serology and/or to perform genomic assessment of HLA class I and class II alleles at low or high resolution (two fields allele assignment) level. This year, the XXX round is being organized. Polish EPT fulfils EFI rules for EPT providers and participants (Tinyurl, 2021).

All laboratories in Poland that serve transplantation purposes use commercially available kits for PCR-SSP and/or PCR-SSO HLA genotyping. NGS technology has been introduced in nine HcL and a few more are currently implementing this technology. SBT technology is employed in three HcL. The real-time PCR technique has been recently implemented in HcL involved in deceased organ donor matching.

Analyses of the past 10 years of Wroclaw’s EPT activity allow to compare results in the following categories: HLA class I serological typing, HLA class I and class II molecular typing at low and high resolution level (for details see Supplementary Figure S1). Starting from the XIX trial, only 2 Polish participants did not comply with the required consensus (none/only one divergent result) due to discrepancies in serological typing of HLA class I antigens (in 2016) and in 2017, due to few discrepancies in results of HLA typing at DNA level (Supplementary Figure S2), including mistyping of a given allele. No discrepant results have been detected in HLA-DRB1 locus genotyping. The improvement in relation to previous rounds of the EPT (Bogunia-Kubik et al., 2006; Bogunia-Kubik and Lange, 2008) confirms the usefulness of participation in EPT. Direct benefits for the laboratory include elimination of incorrect typing of HLA alleles/antigens, reduction in the number of methodological errors, and overall improvement of quality, credibility, and repetitiveness of histocompatibility testing.

Polish HcL may apply for EFI accreditation. Currently, 3 laboratories hold EFI accreditation (from Wroclaw, Poznan and Warsaw) and two others (from Wroclaw and Warsaw) previously accredited by EFI, plan to regain this privilege. Some HcL (their parent entities) also hold various ISO certificates, i.e. 9001:2015, 27001:2017, 45001:2018) or AQAP (2110:2016).

The current EPT system in Poland does not cover procedures other than HLA typing. The interested laboratories participate in EPT schemes for cross-matching, PRA and anti-HLA testing provided by Eurotransplant or INSTAND e.V. and disease association studies (HLA-B27, HLA-DQ2/DQ8) provided by the Institute of Hematology and Blood Transfusion in Prague or INSTAND e.V. (Efi Web, 2023).

One laboratory tests samples provided by ASHI. Six HcL currently participate in the Eurotransplant scheme and 3 in INSTAND EPT. Besides PRA, anti-HLA, anti-C1q, and crossmatching, laboratories participating in the EPT organized by the Eurotransplant Reference Laboratory use the provided samples also for HLA typing. Four laboratories use samples provided by CET or INSTAND in parallel to Wroclaw EPT (Efi Web, 2023).

## Discussion

### Problems faced by Polish HcL

Recent survey and observations from ministerial controls reveal that major problems faced by HcL result from financial constraints. Insufficient number of personnel, inadequate salaries in relation to qualifications and legal responsibility, a whole range of additional office tasks increase the risk of potential laboratory errors and result in a high staff turnover, especially among young diagnosticians at the beginning of their careers. Although appropriate working conditions with adequate separation of processes have been granted as required, suboptimal environmental conditions in office spaces or archives have been reported in some cases. Access to modern equipment is partially financed by MoH, but high costs of its maintenance, regular inspections and servicing covered by parental entities largely affect laboratory’s budget. Maintenance and calibration procedures are gradually improved and better documented, and non-compliance with regular periodical technical inspections of equipment, or the scope of inspections by service providers is less frequently reported. Remote monitoring systems supervise temperature-controlled equipment in most laboratories.

Lack of local EPT providers for procedures other than HLA testing, especially those required for organ transplantation, high costs of participation in international EPTs (registration sample, shipment fees), costs of reagents needed to perform EPT testing, and inconsistency in legal requirements may lead to insufficient control over several procedures. On the other hand, laboratories participating in external EPTs for these procedures face several problems associated with sample quality, which even though remaining beyond control of an EPT organizer (force majeure) may affect overall results. This issue, however, must be urgently formally resolved in the near future. Currently, new contracts are signed only with laboratories participating in EPT.

In general, laboratory methods used in transplantation immunology are well described in medical literature. Dedicated *in vitro* attested commercial kits and reagents are used whenever available. However, several reagents were or are currently expected to be unavailable due to the new *in vitro* Diagnostic Reagent Law - Regulation (EU) 2017/746 (IVDR) (Data Europa, 2017). Lack of access or high costs of IVDR attested reference cells, rabbit complement, and serum controls will probably significantly limit the ability to evaluate patients’ immunization status. Solid phase-based methods face similar problems with IVDR licensing. At this point, however, due to difficulties caused e.g., by the COVID-19 pandemic, the transition periods for the aforementioned regulation have been extended by several years (Eur Lex Europa, 2022).

According to the Transplant Law, all QMS documentation and relevant records must be kept for 30 years from the date of test results delivery. Insufficient or inadequate storage areas and problems with access to electronic documentation for the required period pose another challenge. Since July 2021, all medical data should be stored in dedicated repositories in the form of Electronic Medical Records. High costs associated with the change of laboratory software or its adaptation result in a delay in its implementation. A dedicated platform called e-Transplant, expected to be available in 2024, is planned to cover all elements of the national transplant system and to replace the existing POLTRANSPLANT registries and associated paper documentation.

### Suggestions and perspectives for the future raised by members of the Polish histocompatibility and immunogenetics community

Since the first successful kidney transplant performed in Poland in 1966, surgical techniques have improved, and new methods of organ preservation and better suppressive regimens have been developed. Tremendous developments in immunological diagnostics allowed for the complete replacement of serological HLA typing by molecular methods, monitoring of a recipient’s immunization status with solid phase methods, and crossmatching with higher sensitivity. Shortly, it will be necessary to extend local EPT and/or grant support for participation in external EPT schemes.

None of the above developments or routine operations of HcL will be possible without ensuring further stable funding. Significant funds allocated by MoH support the purchase of modern equipment and introduction of new diagnostic methods in HcL, but salaries in publicly funded entities will have to be gradually increased to prevent high turnover of personnel and an exodus of young diagnosticians. Members of the Polish histocompatibility community hope that the new Act on Laboratory Medicine will both make it easier for young diagnosticians to become specialists in laboratory medical immunology and guarantee them adequate salaries. After all, HcL’s most important resource is well-educated specialists.

In summary, various aspects of obtaining and maintaining quality at Polish HcL, including legal requirements and their fulfillment, supervised by a number of public organizations, have been described in detail. The actual ability to fulfill all requirements is influenced by a variety of factors, both at a general and local level. The common denominator is that promoting the implementation of pro-quality solutions in HcL requires the provision of stable funding.

## Data Availability

The original contributions presented in the study are included in the article/Supplementary Material, further inquiries can be directed to the corresponding author.
