# Supplementary figures and images for: Current status and perspectives of the quality system in histocompatibility laboratories in Poland
Source: Front Genet. 2024 Jan 26;15:1322414. doi: 10.3389/fgene.2024.1322414 (PMC10853346; doi:10.3389/fgene.2024.1322414)

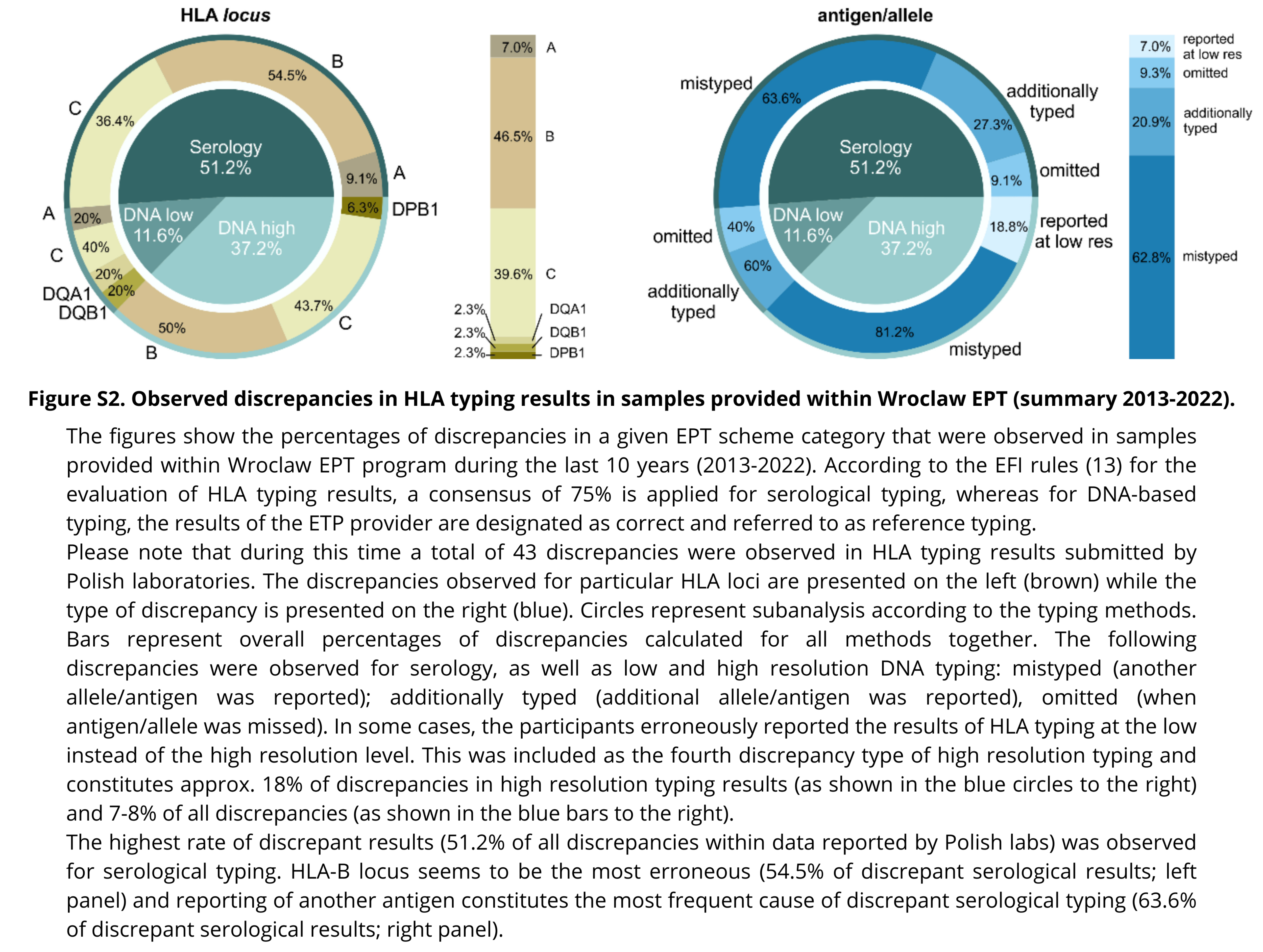

Supplement: Supplementary file 1 [file Image2.jpg]

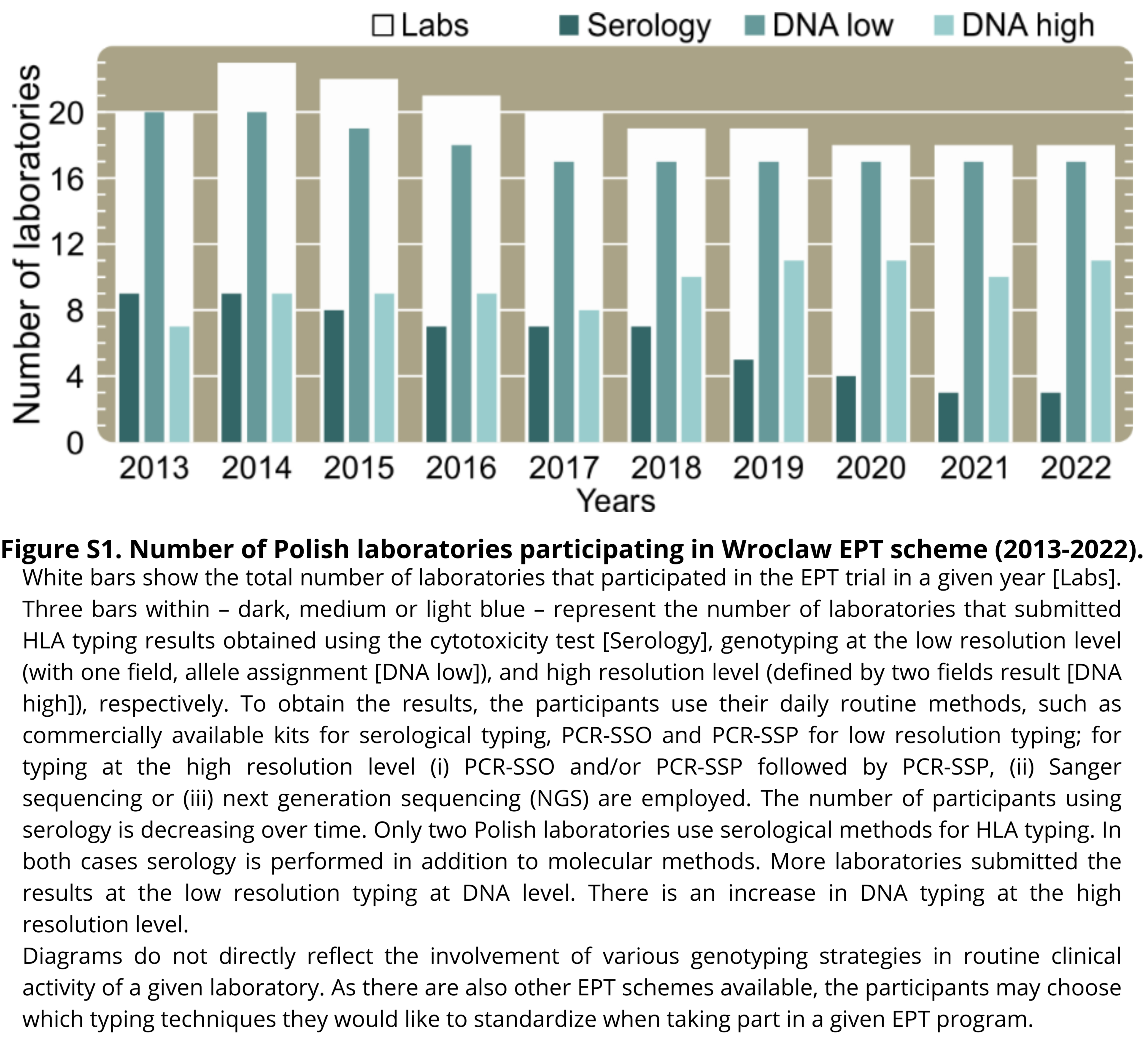

Supplement: Supplementary file 4 [file Image1.jpg]
