# Supplementary material for: Current status and perspectives of the quality system in histocompatibility laboratories in Poland
Source: Front Genet. 2024 Jan 26;15:1322414. doi: 10.3389/fgene.2024.1322414 (PMC10853346; doi:10.3389/fgene.2024.1322414)
Supplement: Supplementary file 2 [file Table1.docx]

**Table S1. The scope of ministerial control at HcL**.

| Scope of ministerial control:   - organizational structure, - personnel, - premises, - laboratory equipment, - reagents, - documentation, - monitoring of processes. |
| --- |
