# Supplementary material for: Current status and perspectives of the quality system in histocompatibility laboratories in Poland
Source: Front Genet. 2024 Jan 26;15:1322414. doi: 10.3389/fgene.2024.1322414 (PMC10853346; doi:10.3389/fgene.2024.1322414)
Supplement: Supplementary file 3 [file Table2.docx]

Table S2. **List of legal requirements that HcL must meet to obtain MoH permission to conduct its activities.**

| Organization:   - - parental entity must be registered in formal register for the health care providers (pl. *Rejestr Podmiotów Wykonujących Działaność Leczniczą*, hereafter *RPWDL*);   - HcL must be registered in *RPWDL* as a medical laboratory (hereafter also ML) or operate within a multispecialty ML registered in RPWDL   - HcL must be registered as ML in the register kept by the professional self-government body (pl. *Krajowa Rada Diagnostów Laboratoryjnych, hereafter KDRL*) or operate within a multispecialty ML registered in the register of KRDL   QMS:   - - SOPs shall describe all aspects of organization and QMS of maternal institution and HcL;   - all laboratory activities must be supervised by institutional Management Representative for Quality, with dedicated personnel within the laboratory responsible for quality issues and adherence to the Transplantation Act;   Personnel:   - - all laboratory directors must be licensed as laboratory diagnosticians, with adequate specialization within the field of medical laboratory immunology, transfusion medicine, hematology, or genetics;   - all medical personnel employed in laboratories dedicated to transplant immunology shall participate in trainings organized by KCBTiK;   Facilities:   - - approval of State District Sanitary Inspector for the laboratory space, confirming that sufficient room and satisfactory conditions have been dedicated to perform laboratory work;   Equipment:   - - all equipment shall be supervised by an appointed person from laboratory staff responsible for every and each apparatus and accompanied by operating manuals, reports from periodical technical supervision, calibration procedures, etc;   Quality control and quality assurance, including proficiency testing:   - - HcL are required to systematically participate in internal and external quality control schemes;   - all nonconformities, as well as corrective and preventive actions must be reported and documented, with nonconformities leading to adverse health effects in either the transplant recipient or donor reported as so-called significant adverse reaction (SAR) under the Transplant Act;   Traceability:   - - HcL must ensure full traceability of test material samples and the documentation associated with those samples;   - HcL must locate and identify all relevant data relating to the quality of the samples of test material and any environmental conditions at the premises, any materials or equipment coming into contact with those samples that pose a presumable risk to their quality. |
| --- |
